# Effective Genome Editing in *Leishmania* (*Viannia*) *braziliensis* Stably Expressing Cas9 and T7 RNA Polymerase

**DOI:** 10.3389/fcimb.2021.772311

**Published:** 2021-11-10

**Authors:** Caroline R. Espada, José Carlos Quilles, Andreia Albuquerque-Wendt, Mario C. Cruz, Tom Beneke, Lucas B. Lorenzon, Eva Gluenz, Angela K. Cruz, Silvia R. B. Uliana

**Affiliations:** ^1^ Departamento de Parasitologia, Instituto de Ciências Biomédicas, Universidade de São Paulo, São Paulo, Brazil; ^2^ Departamento de Biologia Celular e Molecular, Faculdade de Medicina de Ribeirão Preto, Universidade de São Paulo (FMRP-USP), Ribeirão Preto, Brazil; ^3^ Sir William Dunn School of Pathology, University of Oxford, Oxford, United Kingdom; ^4^ Wellcome Centre for Integrative Parasitology, Institute of Infection, Immunity & Inflammation, College of Medical Veterinary and Life Sciences, University of Glasgow, Glasgow, United Kingdom; ^5^ Global Health and Tropical Medicine (GHTM), Instituto de Higiene e Medicina Tropical (IHTM), Universidade de Lisboa (UNL), Lisbon, Portugal; ^6^ Centro de Facilidades para Apoio à Pesquisa, Universidade de São Paulo (CEFAP-USP), São Paulo, Brazil

**Keywords:** CRISPR/Cas9, *Leishmania braziliensis*, PF16, endogenous tagging, knockout, reverse genetics

## Abstract

Until 2015, loss-of-function studies to elucidate protein function in *Leishmania* relied on gene disruption through homologous recombination. Then, the CRISPR/Cas9 revolution reached these protozoan parasites allowing efficient genome editing with one round of transfection. In addition, the development of LeishGEdit, a PCR-based toolkit for generating knockouts and tagged lines using CRISPR/Cas9, allowed a more straightforward and effective genome editing. In this system, the plasmid pTB007 is delivered to *Leishmania* for episomal expression or integration in the β-tubulin locus and for the stable expression of T7 RNA polymerase and Cas9. In South America, and especially in Brazil, *Leishmania* (*Viannia*) *braziliensis* is the most frequent etiological agent of tegumentary leishmaniasis. The *L. braziliensis* β-tubulin locus presents significant sequence divergence in comparison with *Leishmania major*, which precludes the efficient integration of pTB007 and the stable expression of Cas9. To overcome this limitation, the *L. major* β-tubulin sequences, present in the pTB007, were replaced by a *Leishmania* (*Viannia*) β-tubulin conserved sequence generating the pTB007_Viannia plasmid. This modification allowed the successful integration of the pTB007_Viannia cassette in the *L. braziliensis* M2903 genome, and *in silico* predictions suggest that this can also be achieved in other *Viannia* species. The activity of Cas9 was evaluated by knocking out the flagellar protein PF16, which caused a phenotype of immobility in these transfectants. Endogenous PF16 was also successfully tagged with mNeonGreen, and an in-locus complementation strategy was employed to return a C-terminally tagged copy of the *PF16* gene to the original locus, which resulted in the recovery of swimming capacity. The modified plasmid pTB007_Viannia allowed the integration and stable expression of both T7 RNA polymerase and Cas9 in *L. braziliensis* and provided an important tool for the study of the biology of this parasite.

## Introduction

Reverse genetics has been widely employed in studies to characterize the function of protein-coding genes. By manipulating the genome region of interest (ROI), either by removal or modification, one can characterize the resulting phenotypic changes which are indicative of target function. In *Leishmania* parasites, until recently, the function of a wide number of genes was assessed by gene disruption through homologous recombination (Cruz and Beverley, 1990), in which a recombination cassette containing a drug resistance marker and long sequences complementary to the target is delivered to the parasite where it is expected to replace the ROI in the genome (Cruz and Beverley, 1990). The recombination usually occurs in one allele at a time and, thus, more than one round of transfection, with different drug resistance markers, is needed in order to fully replace the ROI. This is an important limitation especially in *Leishmania* parasites in which aneuploidy is an event observed for different species and even strains of the same species (Rogers et al., 2011). In *Leishmania braziliensis*, for example, chromosome 31 is known to be present in four copies. A full knockout of a gene present in this chromosome requires four rounds of transfection with four different resistance markers (Rogers et al., 2011; Lachaud et al., 2014). Moreover, due to high genome plasticity in these organisms, other events such as gene duplication, amplification, alteration of ploidy states, and extrachromosomal elements are not rare, making the complete removal or substitution of a given gene especially challenging (Cruz et al., 1993; Rogers et al., 2011; Iantorno et al., 2017).

The discovery of the CRISPR/Cas (Clustered Regularly Interspaced Short Palindromic Repeats/CRISPR-associated proteins) system as a tool for genome editing was a major breakthrough in molecular biology (Doudna and Charpentier, 2014). In 1987, the CRISPR locus, which contains viral sequences transcribed and processed into individual small RNAs, was discovered in bacteria (Doudna and Charpentier, 2014). Later, in 2010, a family of nucleases, the Cas proteins, was shown to interact with CRISPR RNAs, forming a complex that is capable of recognizing the viral target sequence and cleaving it, protecting the bacteria from a recurrent viral infection and acting like a bacterial immune system (Doudna and Charpentier, 2014). CRISPR/Cas was then purposed as a method for efficient gene knockout in a variety of organisms, and in 2015, it was first employed in *Leishmania* parasites (Sollelis et al., 2015; Zhang and Matlashewski, 2015).

The system requires the expression of a Cas9 nuclease and the presence of a small guide RNA (sgRNA) containing a 20-nt-long protospacer sequence which is complementary to the targeting sequence. The Cas9 forms a complex with the sgRNA and the machinery is guided to the region of interest, placed right after a protospacer-adjacent motif (PAM) sequence (Doudna and Charpentier, 2014). The Cas9–sgRNA complex then binds the target sequence, becoming active and inducing a double-strand break (DSB) 3 nucleotides upstream of the PAM sequence, allowing for a very precise genome editing (Doudna and Charpentier, 2014). In *Leishmania*, this DSB can be repaired through a variety of homology-mediated strategies such as single-strand annealing (SSA), microhomology-mediated end joining (MMEJ), or homology-directed repair (HDR) using a donor DNA cassette to replace the deleted region (Zhang and Matlashewski, 2019). In other organisms, this repair can also be mediated by non-homologous end joining (NHEJ), but this mechanism is absent in *Leishmania* parasites (Passos-Silva et al., 2010; Zhang and Matlashewski, 2015; Zhang et al., 2017; Zhang and Matlashewski, 2019).

Expression of Cas9 as well as sgRNA presence in *Leishmania* can be achieved in different ways. In 2015, Sollelis et al. (2015) used two plasmids, one for Cas9 expression and the other carrying a sgRNA under control of an U6 promoter and the donor DNA. Both plasmids were transfected at once, and the knockout of the paraflagellar rod 2 locus in *Leishmania major* was obtained without off-target effects. Zhang and Matlashewski (2015), using a single plasmid to drive both Cas9 expression and sgRNA transcription under a RNA polymerase I promoter, successfully deleted the A2 multigene family (multicopy genes) and the miltefosine transporter in *Leishmania donovani*. In addition, this system was effective also for endogenous gene tagging and point mutation insertion (Zhang and Matlashewski, 2015; Bryant et al., 2019).

Later, Beneke et al. (2017) developed LeishGEdit, a toolbox for genome editing in *Leishmania*, in which Cas9 and T7 RNA polymerase coding sequences are delivered to the parasite for episomal expression in a plasmid or as a linear cassette for integration in β-tubulin locus. Generation of sgRNA templates and donor DNAs is accomplished by PCR using primers retrieved by the LeishGEdit platform upon Gene ID input (Beneke and Gluenz, 2019). Using this system, genes were successfully deleted in a variety of *Leishmania* species including *Leishmania mexicana* (Beneke et al., 2017; Grewal et al., 2019; Shrivastava et al., 2019; Tupperwar et al., 2019; Burge and Damianou, 2020; Damianou et al., 2020), *L. major* (Beneke et al., 2017; Damasceno and Reis-Cunha, 2020; Espada et al., 2021), *Leishmania tarentolae* (Turra et al., 2021), *L. donovani* (Martel et al., 2017), and *L. braziliensis* (Adaui et al., 2020). Except for *L. major* (Beneke et al., 2017; Espada et al., 2021), in all other cases, Cas9 and T7 RNA polymerase were expressed *via* an episome, requiring the maintenance of drug selection to avoid plasmid loss. Integration allows for maintenance of Cas9 expression in circumstances where drug selection is unfeasible if further genome editing is necessary, for example, in experiments using sandflies and mice. Moreover, due to the presence of an active RNAi machinery in *L. braziliensis*, the presence of an episomal vector can be potentially deleterious to the parasite (Lye et al., 2010). Importantly, it was shown in *L. major* that stable expression of Cas9 integrated in β-tubulin locus has no effect on parasite growth (Beneke et al., 2017).

In the pTB007 plasmid, the homology arms designed to promote cassette integration in the β-tubulin locus are about 350-nt-long sequences derived from the *L. major* genome (Beneke et al., 2017). The comparison of these sequences with the corresponding *L. braziliensis* β-tubulin intergenic regions revealed a degree of identity of 69.0% and 72.9% for 5′ and 3′ homology sequences, insufficient to allow integration of this cassette by homologous recombination in this species. Since *L. braziliensis* is the most important causative agent of tegumentary leishmaniasis in Brazil (Grimaldi et al., 1987; Silveira, 2019), we set out to modify the plasmid, including a *L. braziliensis* conserved β-tubulin sequence, allowing for the integration of this cassette in this species and potentially in other species of the *Viannia* subgenus. We successfully generated a *L. braziliensis* lineage stably expressing functional Cas9 and T7 RNA polymerase proteins allowing the generation of knockout, tagged, and complemented lines using CRISPR/Cas9-mediated genome editing.

## Materials and Methods

### Parasites

The cell lines used in this work were *L. braziliensis* M2903 (MHOM/BR/75/M2903) and its genetically modified derivatives. Promastigotes were cultivated at 25°C in M199 medium (Sigma-Aldrich) supplemented with 2.2 g/L NaHCO_3_, 0.005% hemin, 40 mM 4-(2-hydroxyethyl)piperazine-1-ethanesulfonic acid (HEPES) pH 7.4, 10% heat-inactivated fetal calf serum (FCS) (Gibco™), and 0.2 µg/ml biopterin (Sigma-Aldrich). The appropriate selection drugs were added at 32 µg/ml hygromycin B (Gibco™), 5 µg/ml blasticidin S hydrochloride (Gibco™), and 20 µg/ml puromycin dihydrochloride (Gibco™).

### Replacement of pTB007 Homology Arm Sequences

*Leishmania braziliensis* β-tubulin 5′ and 3′ regions chosen for cloning into pTB007 were amplified from *L. braziliensis* M2903 reference strain using the primer pairs 5′*Hin*dIII-β-tub_F plus 5′*Spe*I-β-tub_R and 3′*Bsi*WI-β-tub_F 3′*Sbf*I-β-tub_R, respectively. The sequences of all primers used in this study are presented in **Supplementary Table 1**.

The plasmid pTB007 (Beneke et al., 2017) and the amplified 5′ β-tubulin fragment were digested with *Hin*dIII and *Spe*I overnight at 37°C and purified. 5′ β-tubulin was then ligated to pTB007 using T4 DNA Ligase (New England Biolabs). Correct insertion was verified by restriction enzyme digestion with *Fse*I. The obtained plasmid and 3′ β-tubulin PCR product were then digested with *Sbf*I and *Bsi*WI overnight. Correct insertion was verified by restriction enzyme digestion with *Fse*I and *Sgr*AI. For phosphoglycerate kinase B 5′ UTR (PGBK5′) re-insertion upstream Cas9 in the plasmid, PGBK5′ was amplified from pTB007 with the primer Repair_Rev and Repair_Fow_*Spe*I. pTB007_Viannia and PGKB5′ PCR product were digested with *Spe*I overnight and ligated using T4 DNA Ligase (New England Biolabs). The pTB007_Viannia plasmid was sequenced using the primers 5′*Hin*dIII-β-tub_F, Seq-VpTB007-A_R, Seq-VpTB007-A_F, Seq-VpTB007-B_F, Seq-VpTB007-B_R, Seq-VpTB007-C_F, and Seq-VpTB007-C_R at Source BioScience Sequencing Oxford, UK.

The plasmid pTB007_Viannia was linearized with *Hin*dIII and *Sbf*I and purified and 6 µg was transfected into 2 · 10^7^ *L. braziliensis* M2903 log-phase promastigotes as described in Section 2.4.

### Generation of Guide RNAs and Donor DNAs

Knockouts and tagged mutants used in this work were generated using the LeishGEdit toolkit (Beneke et al., 2017; Beneke and Gluenz, 2019). The PF16 gene ID LBRM2903_200071200 was used to retrieve from the LeishGEdit (http://www.leishgedit.net/) website the primers for the generation of sgRNA templates (PF16_5′sgRNA and PF16_3′sgRNA) and donor DNAs (PF16_UFP, PF16_URP, PF16_DFP, and PF16_DRP) through PCR amplification (**Supplementary Table 1**). For the generation of add-back lines, 5′ and 3′ sgRNAs target sequences were designed using the EuPaGDT (Peng and Tarleton, 2015) website and replaced in PF16_5′sgRNA and PF16_3′sgRNA generating PF16_AB_5′sgRNA and PF16_AB_3′sgRNA primers, respectively.

The sgRNA templates and donor DNAs were generated by PCR using Phusion^®^ High-Fidelity DNA Polymerase (New England Biolabs) according to the specifications of Beneke and Gluenz (2019). For PF16 knockout, pTBlastv1 (Beneke et al., 2017) was used as template together with the UFP and DRP primers, and for PF16 tagging at the C-terminus, pPLOTv1 puro-mNeonGreen-puro (Beneke et al., 2017) was used together with DFP and DRF primers (Beneke et al., 2017). Donor DNA for complementation purposes was generated using Lb C9/T7 PF16::mNG cell line genomic DNA, extracted using DNeasy Blood & Tissue Kit (Qiagen), as template for PCRs. Amplification of this region was done with the primer pairs Lb_PF16_UTR-F and Lb_PF16_UTR-R using Phusion^®^ High-Fidelity DNA Polymerase (New England Biolabs). The band correspondent to the tagged PF16 cassette was gel purified using NucleoSpin Gel and PCR Clean-up (NucleoSpin).

PCR-generated 5′ and 3′ sgRNAs were pooled together with donor DNA for the generation of knockout and complemented lines. For PF16 C-terminus tagging, the 3′ sgRNA template was pooled together with donor DNA. After precipitation, DNA was sterilized by incubation at 94°C for 5 min.

### Transfection and Cloning

Transfections were performed using 1 · 10^7^ *L. braziliensis* M2903 Cas9/T7 log-phase promastigotes according to the protocol described by Beneke and Gluenz (2019). For cloning, after three splits at 1:100 (culture: M199) ratio, 500 parasites were spread on M199 1% agar plates containing 1.2 µg/ml biopterin (Sigma-Aldrich) and the appropriate selection drug (see Section 2.1). At least three clones of knockout and complemented transfectants were collected and transferred to M199. Alternatively, Lb C9/T7 and tagged transfectants (PF16::mNeonGreen) were not cloned since double-allele replacement was not mandatory.

### DNA Extraction and Confirmative PCRs

For PCR verification of genomic modifications, DNA was extracted using the protocol previously described by Rotureau et al. (2005).

To confirm pTB007_Viannia cassette integration in β-Tub locus, primers annealing outside the target region (5′ Tub-F and 3′ Tub-R), inside Cas9 CDS (Cas9-F and Cas9-R), and inside the hygromycin resistance gene (*Hyg-F*) were used. Primers annealing to the sequence encoding the TATA box-binding protein were used as an internal control (*tbp*-F and *tbp-*R). PCRs were done using *Taq* Polymerase (Sinapse).

PF16 di-allelic (double) knockouts were verified through PCRs using a primer annealing at PF16 3′ UTR (Lb_PF16_UTR-R) and primers annealing inside PF16 CDS (PF16-F) or inside the blasticidin resistance gene (*Blast-F*). For complementation verification, PCRs using these same primers plus one annealing inside the puromycin resistance gene (*Puro-F*) were performed. These PCRs were done using GoTaq^®^ DNA Polymerase (Promega).

### Western Blotting

Approximately 2 · 10^7^ log-phase promastigotes were harvested and washed with PBS 1× cOmplete^TM^ protease inhibitor cocktail (Roche). Parasites were incubated with lysis buffer (50 mM Tris–HCl 1 M pH 7.4, 2% SDS, 1 mM PMSF, 1× cOmplete^TM^) and boiled for 10 min followed by incubation on ice for 5 min. Protein extract (30 µg) was separated by sodium dodecyl sulfate-polyacrylamide gel electrophoresis (12% separation and 5% stacking gel) and electrotransferred to 0.45 µM Amersham^TM^ Protran^®^ nitrocellulose membranes (GE). The membrane was blocked with 5% milk at room temperature for 1 h. After that, the membrane was incubated with the primary anti-flag M2 antibody (F1804-MG Sigma-Aldrich) in 1% milk-TBST solution (20 mM Tris, 150 mM NaCl, 0.1% Tween 20 pH 7.6) at 1:4,000 (antibody:solution) for 1 h. After washing, the membrane was incubated with the secondary anti-mouse NA931V antibody (GE Healthcare) in a TBST solution without milk at 1:10,000 (antibody:solution) for 1 h. Detection was performed with ECL™ Prime Western Blotting System (Cytiva Amersham^TM^).

### Growth Curve

Parasites were seeded at the density of 2 · 10^5^ promastigotes/ml in M199 media without hygromycin and counted in a Neubauer chamber daily for 7 days. Three biological replicates were evaluated.

### *In-Vitro* Infection of THP-1 Cells

THP-1 cells were cultured in complete RPMI-1640 medium (Sigma-Aldrich) supplemented with 10% heat-inactivated fetal bovine serum (Sigma-Aldrich) and 0.5% penicillin–streptomycin (Thermo Fisher Scientific) at 37°C and a 5% CO_2_ atmosphere. THP-1 monocytes were seeded on glass coverslips in 24-well plates and differentiated into macrophages by incubation with 30 ng/ml 4α-phorbol 12-myristate 13-acetate (PMA) (Sigma-Aldrich) for 24 h (Daigneault et al., 2010). Non-differentiated cells were removed, and fresh RPMI-1640 medium was added. The cells were further incubated for 48 h for complete maturation and improvement of the phagocytic properties (Gatto et al., 2020). For the infection, metacyclic promastigotes were obtained from stationary *Leishmania* cultures by the Ficoll PM 400 (Cytiva) density gradient assay as described by Spath and Beverley (2001) and Ruy et al. (2019). At this point, metacyclogenesis rates were determined by counting the parasites before and after the Ficoll purification. The number of purified metacyclics was divided by the number of total parasites to obtain the percentage of metacyclic promastigotes for Lb WT and Lb C9/T7. THP-1 macrophages were then infected with *L. braziliensis* metacyclic promastigotes at the MOI of 10:1 (parasites:macrophage) at 33°C in 5% CO_2_ atmosphere for 4 h. After that, non-internalized parasites were removed by washing and fresh RPMI-1640 medium was added. Plates were kept in fresh medium at 33°C and 5% CO_2_ atmosphere for 24, 48, and 72 h. Cells were fixed with PBS:methanol (1:1 v/v) solution for 10 min and stained for 45 min with 10× Giemsa solution. The percentage of infected macrophages, number of amastigotes per infected macrophage, and infectivity index (percentage of infected macrophages multiplied by the average number of amastigotes per macrophage) were determined by counting 100 cells per coverslip. The experiment was performed twice in technical triplicates.

### Microscopy

Parasites were incubated in PBS with 10 μg/ml Hoechst 33342 (Invitrogen), centrifuged at 800*g* for 10 min, suspended in PBS–glucose, and applied onto a microscope slide. Living cells were immediately imaged and filmed in a Leica DMI6000B microscope with a HC PL APO 100×/1.40 oil immersion objective and a DFC365FX-624202712 camera. The filters used for Hoechst 33342 and mNeonGreen were A4 360/470 nm (excitation/emission) and L5 480/527 nm (excitation/emission), respectively. As background control, parental Lb C9/T7 strain was imaged using specific filters at the same exposure time. Videos for flagella movement quantification were acquired in the same microscope with a HC PL FLUOTAR L 40×/0.60 dry objective at approximately 20 frames per second (fps). Alternatively, videos illustrating the phenotype of motility were acquired in a Carl Zeiss LSM 780 AxioObserver microscope with a Plan-Apochromat 63×/1.40 Oil DIC M27 objective. Images and movies were processed using Fiji (Schindelin et al., 2012).

The flagella tip movement was tracked in individual parasites (15 parasites evaluated in four to five fields) using the Manual Tracking plugin inside the Fiji software (31), and the velocity (µm/s) and distance (µm/frame) values were calculated using the *XY* coordinates from the tip of each flagellum in 100 consecutive frames acquired under 20 fps. Then, the velocity and the dislocation from 15 parasites of each field were averaged and plotted on GraphPad Prism 8.

### Statistical Analysis

Statistical analyses were performed using non-parametric *t*-test (Mann–Whitney) and one-way ANOVA followed by Tukey’s multiple comparison tests using GraphPad Prism 8. Statistical significance was considered when *p*-value <0.05.

## Results

### Generation and Characterization of *Leishmania braziliensis* M2903 Cas9/T7 Cell Line

**Figure 1:**
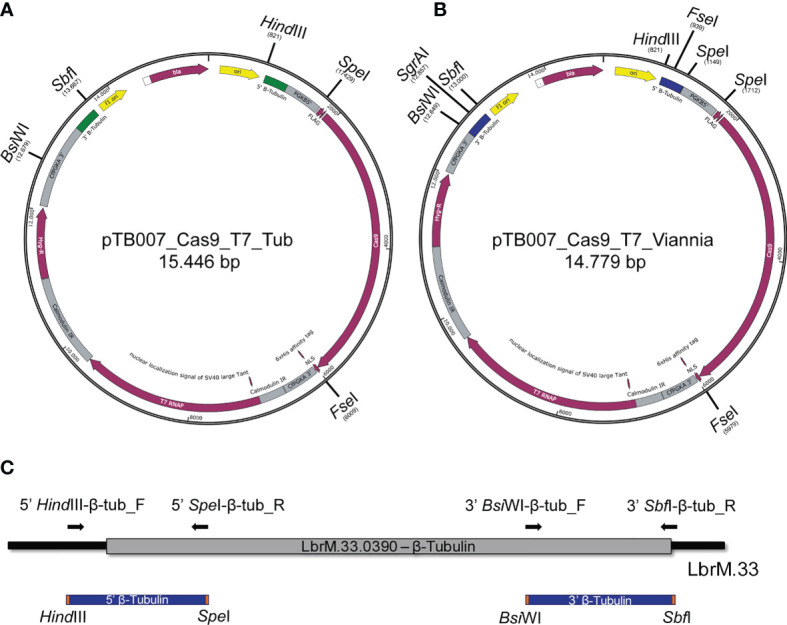
Replacement of *Leishmania major* β-tubulin sequences in pTB007 by *Leishmania braziliensis* β-tubulin conserved sequences. **(A)** Original plasmid for episomal expression of Cas9 and T7 RNA polymerase in *Leishmania* spp. and for the integration and stable expression of these proteins in *L. major* and *Leishmania mexicana* (12). The restriction enzymes used for directional cloning were *Bsi*WI and *Sbf*I for replacement of 3′ end (green) and *Hin*dIII plus *Spe*I for replacement of 5′ end (green). **(B)** *Leishmania major* sequences [represented in green in **(A)**] were replaced by *L. braziliensis* sequences [represented in blue in **(B)**] allowing the integration of this cassette in this species for the stable expression of Cas9 and T7 RNA polymerase. PGKB5′ that was removed in the first cloning step was returned to the plasmid by directional cloning using the enzyme *Spe*I. **(C)** Schematic representation of the *L. braziliensis* β-tubulin sequences chosen for replacement in pTB007 (blue). Primers used to amplify the target region are represented as black arrows.

To enable the integration of the pTB007 cassette in *L. braziliensis* M2903 for the stable expression of Cas9 and T7 RNA polymerase in this cell line, the *L. major* 5′ 344-bp and the 3′ 358-bp homology arms of pTB007 plasmid were replaced by a *L. braziliensis* conserved region of 322 bp in the 5′ and of 345 bp in the 3′ end of β-tubulin coding sequence by directional cloning (**Figures 1A–C**). The strategy used to replace these fragments in pTB007 plasmid caused the loss of PGKB5′ which was again amplified from pTB007 and returned to pTB007_Viannia also by directional cloning. The correct assembly of pTB007_Viannia was confirmed through RFLP after digestion with *Fse*I and *Sgr*AI and through sequencing of the modified regions. *In silico* analysis using BLAST (Altschul et al., 1990) revealed a high percentage of identity of the chosen homology arms (above 90%) with other *Leishmania* species of *Viannia* and *Leishmania* subgenus potentially enabling its integration in these species (**Table 1**).

**Table 1:** *In silico* basic local alignment of *Leishmania braziliensis* 5′ and 3′ β-tubulin sequences cloned in pTB007_Viannia.

Strain^a^	5′ β-tubulin			3′ β-tubulin			Accession number^e^
	Query cover^b^	E-value^c^	Per. ident^d^	Query cover^b^	E-value^c^	Per. ident^d^	
*Leishmania braziliensis* M2904	100%	6.00E-166	100.00%	98%	6.00E-176	100.00%	LS997620.1
*Leishmania peruviana* PAB-4377_V1	100%	5.00E-147	100.00%	98%	6.00E-176	100.00%	LN609230.1
*Leishmania guyanensis* strain M4147	73%	6.00E-111	97.90%	98%	1.00E-172	99.41%	DQ836297.1
*Leishmania amazonensis* strain UA301	91%	1.00E-107	91.03%	98%	1.00E-163	97.94%	CP040136.1
*Leishmania mexicana* strain U1103	91%	1.00E-107	91.03%	98%	6.00E-166	98.24%	XM_003875406.1
*Leishmania major* strain Friedlin	93%	2.00E-106	90.46%	98%	1.00E-158	97.05%	FR796404.1
*Leishmania infantum* strain TR01	93%	2.00E-105	90.13%	98%	3.00E-164	97.94%	CP027820.1
*Leishmania donovani* strain LdCL	94%	8.00E-105	90.16%	98%	6.00E-166	98.24%	CP029507.1
*Leishmania tarentolae* strain UC	73%	1.00E-87	92.02%	98%	5.00E-157	96.76%	DQ309033.1

a Leishmania spp. and strain code retrieved in BLAST (36) search.

b Percentage of the original sequence aligned to the retrieved sequence.

c Expectation value measures the likelihood of the alignment arising by chance instead of as a result of true correspondence.

d Percentage of identity of the compared sequences.

e Accession number of the retrieved sequences in GenBank.

**Figure 2:**
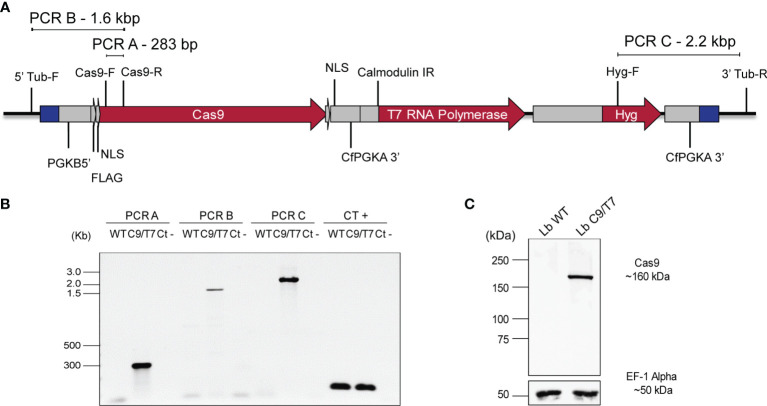
Confirmation of pTB007_Viannia cassette integration and Cas9 expression in *Leishmania braziliensis* M2903 Cas9/T7 lineage (Lb C9/T7). **(A)** Schematic representation of pTB007_Viannia cassette after integration in β-tubulin locus on chromosome 33 (black line) and primers used in each PCR to confirm the integration and correct orientation of this cassette in the transfectants (Lb C9/T7). **(B)** The presence of Cas9 CDS in the transfected population was confirmed using the primer pair Cas9-F and Cas9-R (PCR-A) which amplified a 283-bp fragment in Lb C9/T7 but not in Lb WT cell line. The correct orientation of the cassette in the β-tubulin locus was confirmed using the primer pairs—5′ Tub-F and Cas9-R (PCR-B) in the 5′ end and Hyg-F and 3′ Tub-R (PCR-C) in the 3′ end—and amplified fragments of 1.6 and 2.2 kb, respectively, only in the C9/T7 line. To confirm the presence of DNA in the reactions, a positive control PCR (CT +) was done using the primer pair *tbp*-F and *tbp*-R which anneals in the TATA box-binding protein CDS. **(C)** Expression of Cas9 in the transfectant line (Lb C9/T7) and in the WT (Lb WT) was assessed through Western blotting using an anti-FLAG antibody. The presence of a ~160-kDa band relative to the Cas9 protein fused to a FLAG tag was observed only in Lb C9/T7 confirming the expression of this protein in this cell line. The loading control was done using an anti-EF-1-Alpha antibody which revealed a ~50-kDa band in both Lb WT and Lb C9/T7 protein extracts.

The pTB007_Viannia plasmid was linearized and transfected into *L. braziliensis* M2903 for integration in the β-tubulin locus and expression of Cas9 and T7 RNA polymerase in these parasites. *In silico* analysis revealed possible integration of this cassette in 28 loci in the *L. braziliensis* genome, 24 of them in chromosome 33, where β-tubulin genes are tandemly organized, three of them in chromosome 8, and one in chromosome 21. All regions retrieved as possible recombination locus are annotated as β-tubulin coding sequences. Successful integration in *L. braziliensis* would most likely have happened on one of the chromosome 33 β-tubulin gene copies given the higher number of copies on this chromosome compared with the other loci (chromosomes 8 and 21). Cassette integration was therefore verified by PCR using specific primers targeting β-tubulin gene copies present in chromosome 33 (**Figures 2A, B** **)**. The 283-bp fragment amplified in PCR-A indicated the presence of Cas9 open reading frame in the transfectant *L. braziliensis* M2903 C9/T7 (Lb C9/T7) but not in the wild-type parasite (Lb WT). The amplification of 1.6- and 2.2-kbp fragments in PCRs B and C, respectively, in Lb C9/T7 revealed the correct orientation of this cassette in chromosome 33 (**Figure 2B**). We cannot exclude though that integration might have happened in multiple loci. Cas9 expression in Cas9/T7 transfectant was confirmed by Western blotting with anti-FLAG which revealed the presence of a ~160-kDa band corresponding to the Cas9 protein fused to a FLAG tag (**Figure 2C**).

**Figure 3:**
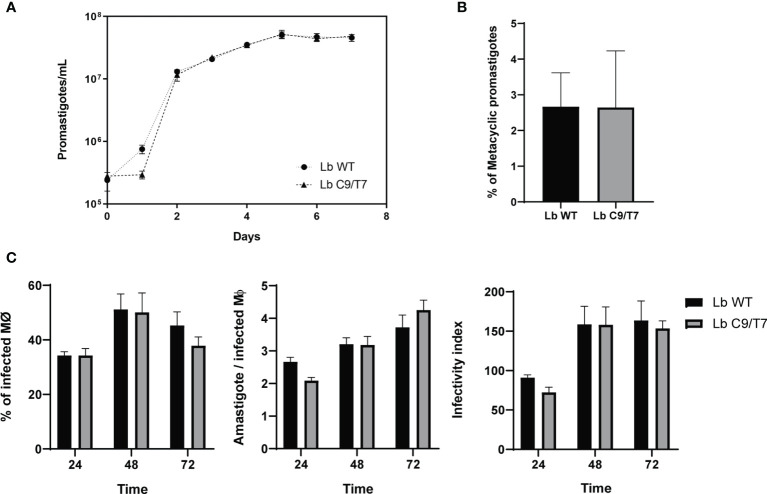
Phenotypic characterization of Lb C9/T7 and Lb WT growth in culture, metacyclogenesis, and infectivity in THP-1 cell lines. **(A)** Parasite density was adjusted to 2 · 10^5^ promastigotes/ml and followed up in three biological replicates during 7 days. No significant difference in parasite growth in culture was observed when Lb C9/T7 and Lb WT area under the curves were compared (*t*-test; *p* < 0.05). **(B)** Culture density was adjusted to 2 · 10^5^ promastigotes/ml. After 7 days, parasites were counted in biological triplicates before and after Ficoll enrichment to determine the percentage of metacyclics in Lb WT and Lb C9/T7, and no significant differences were observed (*t*-test; *p* < 0.05). **(C)** Infectivity of Lb C9/T7 and Lb WT to THP-1-differentiated macrophages was compared after 24, 48, and 72 h. THP-1 cells were infected with Ficoll-purified metacyclic promastigotes at the MOI of 10:1 (metacyclics:cell) during 4 h at 37°C 5% CO_2_. Infections progressed during the selected time points, and the percentage of infected macrophages, the number of amastigotes per macrophage, and the infectivity index were determined by counting 100 Giemsa-stained cells per slide. The graphs are representative of two independent experiments performed in technical triplicates, and no significant difference between Lb WT and Lb Cas9/T7 was observed (*t*-test; *p* < 0.05).

The effects of Cas9 and T7 expression on growth and infectivity of *L. braziliensis* M2903 Cas9/T7 cell line were evaluated in order to ensure that the integration of this cassette would not cause significant fitness loss in this cell line compared with the Lb WT. No significant differences were observed when Lb C9/T7 and Lb WT growth curves were compared (**Figure 3A**). Infectivity to macrophages was also evaluated in THP-1 cells infected with metacyclic promastigotes, and no significant differences were observed when Lb C9/T7 was compared to Lb WT (**Figure 3C**). The metacyclogenesis rate was also evaluated before infection through comparison of populations before and after Ficoll enrichment, and no significant differences were observed between Lb WT and Lb C9/T7 (**Figure 3B**).

### Functional Validation of Cas9 and T7 RNA Polymerase Activity in *Leishmania braziliensis* M2903 Cas9/T7

**Figure 4:**
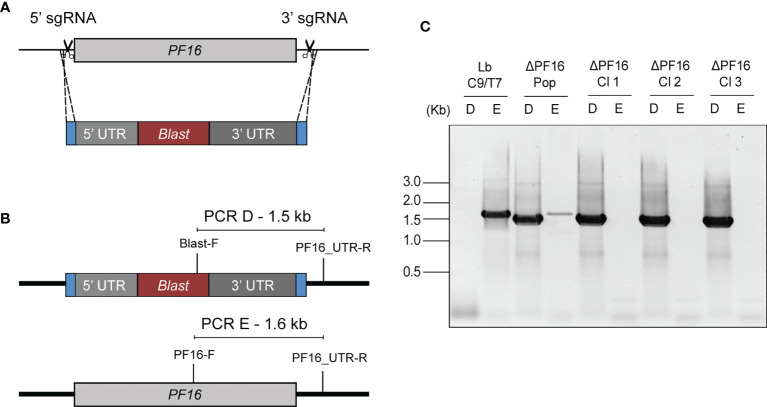
Schematic representation of the strategy used for *PF16* knockout in Lb C9/T7 and for PCR screen of transfectants. **(A)** Two sgRNAs, one at the 5′ end and the other at the 3′ end of the CDS (scissors), were used to direct Cas9 for the complete removal of PF16 CDS. A donor DNA containing a blastidicin resistance marker (red) and a 30-nt homology arm at each end (blue) were also delivered to mediate a double-strand break repair. **(B)** Two PCRs (PCR-D and PCR-E) were performed in order to screen for the presence or absence of PF16 (PCR-E) and blasticidin resistance marker (PCR-D) in the parental line (Lb C9/T7), in the transfectant population (ΔPF16-Pop), and in the clones ΔPF16 Cl1 to 3. **(C)** Electrophoresis in 1% agarose gel shows amplification of a 1.6-kb fragment, relative to the presence of PF16 CDS (E), only in the parental line (Lb C9/T7) and in the non-cloned population (ΔPF16-Pop).

The effectiveness of the Cas9/T7 system was tested by knocking out the *PF16* gene (LBRM2903_200071200) which encodes a protein that composes the axoneme central apparatus essential for flagella motility. For that, a pair of sgRNAs, each one cleaving at one end of *PF16*, were used, together with a donor DNA cassette containing a blasticidin resistance marker (pTBLAST) for homology-mediated DSB directed repair (**Figure 4A**). Fifteen days post-transfection, the negative control was completely dead and the knocked out population (ΔPF16) arose in the culture. After cloning in M199 agar plates, a total of three clones were screened for knockout confirmation by two PCRs. One reaction was done with a forward primer annealing inside PF16 CDS and the other with a primer annealing inside the blasticidin resistance marker both of them using a common reverse primer annealing outside PF16 CDS (**Figure 4B**). Amplification of PF16 was observed only in the parental line (Lb C9/T7) and in the non-cloned transfectant population confirming that all collected clones were di-allelic knockouts (**Figure 4C**). Amplification using forward primers annealing inside the blasticidin resistance marker was used as a positive control of DNA integrity in di-allelic knockouts (**Figure 4C**).

### Tagging of Endogenous PF16 in Lb C9/T7 and in Locus PF16 Complementation

**Figure 5:**
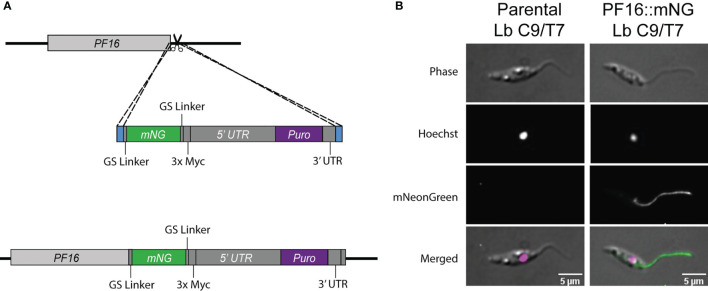
Schematic representation of the strategy used for tagging endogenous PF16 in Lb C9/T7 and localization of PF16 in the transfectants. **(A)** One sgRNA (scissors) was used to direct Cas9 for a DSB at *PF16* 3′ UTR. A donor DNA containing a mNG CDS (green) and a puromycin resistance marker (purple) and 30 nt long homology arms at each end (blue) were also delivered to mediate DSB repair. **(B)** Localization of fused PF16::mNeonGreen (PF16::mNG) in *Leishmania braziliensis* C9/T7. The left column shows images acquired from the parental line (Lb C9/T7), and the right column shows images acquired from the tagged line (Lb C9/T7 PF16::mNG). Each row shows phase contrast, Hoechst (DNA), mNG (tagged CDS), and merged channels, respectively.

The Cas9 activity in Lb C9/T7 cell line was also confirmed through tagging of endogenous PF16 with the reporter protein mNeonGreen (mNG). In this case, only one sgRNA targeting the 3′ end of PF16 CDS was delivered to Lb C9/T7 together with a cassette for DSB repair containing the CDS of mNG fluorescent protein and the puromycin resistance marker (**Figure 5A**). Imaging of the recovered transfectants revealed an intense signal all over the flagella and the flagellar pocket in Lb C9/T7 PF::mNG transfectant and a complete absence of signal in parental Lb C9/T7 (**Figure 5B**). Moreover, tagged parasites retained the motility and swimming capacity and were able to dislocate in culture.

**Figure 6:**
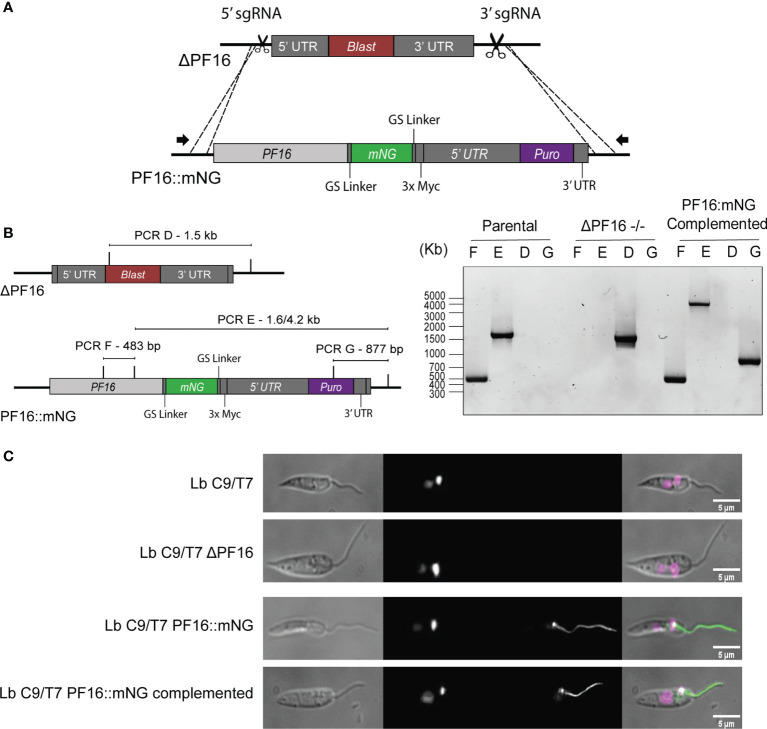
Schematic representation of the in-locus complementation strategy. **(A)** Two sgRNAs (scissors) at the 5′ and 3′ ends of the pTBLAST cassette insertion sites were used to direct Cas9-mediated DSB in Lb C9/T7 ΔPF16 cell line. A donor DNA amplified from Lb C9/T7 PF16::mNG transfectant containing the PF16 CDS fused to mNG (green) and a puromycin resistance marker (purple) was also delivered to mediate DSB repair. **(B)** Four PCRs (PCR-D, PCR-E, PCR-F, and PCR-G) were performed to screen for the presence or absence of PF16 CDS (PCR-E and PCR-F) and blasticidin resistance gene (PCR-D) in the parental line (Lb C9/T7), in the PF16 di-allelic knockout line (ΔPF16−/−), and in the complemented cell line. Electrophoresis in 1% agarose gel showed the amplification of a 483-bp fragment (PCR-F) and a 1.6- or 4.2-kb fragment (PCR-E) in parental and add-back lines, respectively, relative to the presence of PF16 CDS. Amplification using a forward primer annealing in the blasticidin resistance marker was only observed in the genomic DNA of the knockout cell line, and amplification using a forward primer annealing inside the puromycin resistance gene was only observed in the complemented cell line. **(C)** Imaging of cell lines generated in this study: parental (Lb C9/T7), knockout Lb C9/T7 ΔPF16, tagged (Lb C9/T7 PF16::mNG), and complemented (Lb C9/T7 PF16::mNG complemented). Each column shows phase contrast, Hoechst (DNA), mNeonGreen (tagged CDS), and merged channels, respectively.

The PF16 tagged cell line was then used for a strategy called in-locus complementation, in which the PF16 CDS was returned to the original locus from where it was removed. The genomic DNA of Lb C9/T7 PF16::mNG cell line was extracted and used as template for the amplification of the donor DNA cassette containing the PF16 CDS fused to mNG and also carrying the puromycin resistance marker. Two sgRNA templates, one targeting 5′ and the other at 3′ of the pTBLAST cassette present in ΔPF16, were delivered to the cell together with the repair cassette containing PF16::mNG and puromycin resistance marker (**Figure 6A**). After cloning, parasites were screened for the absence of pTBLAST cassette and the presence of PF16 and puromycin resistance marker (**Figure 6B**).

Using primers annealing outside PF16 CDS in combination with primers annealing inside blasticidin, puromycin and *PF16* genes we were able to confirm that PF16 was returned to the original locus (**Figures 6A, B** **)**. The absence of amplification in PF16::mNG-complemented transfectants using primers annealing inside blasticidin resistance gene confirmed that the substitution was achieved in all alleles. As expected, amplification of PF16 was confirmed in parental and complemented lines but not in the ΔPF16 (**Figure 6B**). The differential amplicon size in PCR-E using primers annealing inside PF16 and in its UTR in parental and complemented lines confirmed the integration of the entire cassette amplified from the tagged line (**Figure 6B**). Additionally, microscopy showed that the complemented transfectants presented an endogenously tagged PF16 with a localization pattern matching the observed for the tagged line (**Figure 6C**).

### Phenotypic Analysis of Generated Transfectants

**Figure 7:**
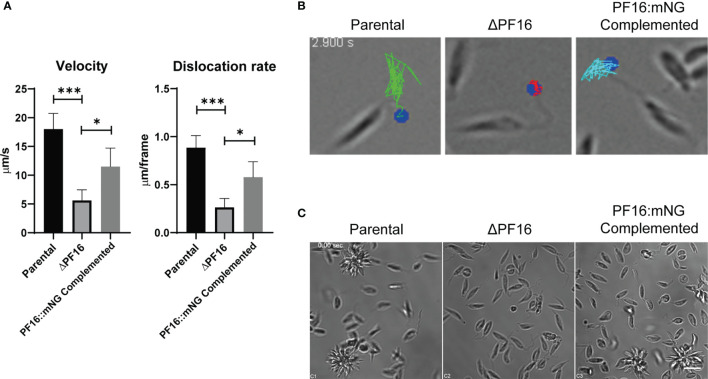
Phenotypic characterization of parental, PF16 knockout, and complemented lines. **(A)** Parasites were seeded in microscopy slides and imaged at 20 frames per second. Flagella tip was tracked in individual parasites, and mean velocity and dislocation rates per frame were estimated for parental (Lb C9/T7), knockout (Lb C9/T7 ΔPF16), and complemented (Lb C9/T7 PF16::mNG) lines. Each graph bar represents the mean ± SD of 15 parasites evaluated in four to five focal fields. *p < 0.05, ***p < 0.0003. **(B)** Trajectory of the tip of the flagella (dot—flagella tip, white line—overlay of the tracked paths). Scale bar 5 µm. For the movie file, see **Supplementary Video 2**. **(C)** Filming each cell line revealed the presence of the flagella and unchanged morphology of genetically modified cell lines. Motility was impaired in knocked out parasites compared with the parental line, and parasites remained at the same place all over the acquisition. In complemented and in parental lines, it was possible to see swimming parasites entering the focal field occasionally. Scale bar 10 µm. For movie files, see **Supplementary Video 1**.

Since PF16 is known to be involved in flagellar motility, we assessed whether or not the genome editing strategy employed herein resulted in an expected phenotype of loss of motility in ΔPF16 and recovery when PF16 was complemented. Parasites were seeded in culture dishes in the presence of PBS–glucose and left to decant for 20 min, in order to avoid the fluid movement to bias the moving analysis. Each cell line was imaged for 30 s and flagella motility and dislocation over the acquired frames were analyzed. While the parental line Lb C9/T7 was able to actively swim and dislocate, knocked out parasites concentrated at the bottom of the slide were not able to swim around the field. In complemented cell lines, the capacity for swimming and dislocating in the supernatant was restored (**Figure 7C** and **Supplementary Video 1**). Analysis of individual parasites in each cell line (15 parasites/field) was done in order to estimate flagella beating velocity and dislocation. When PF16 is deleted from *Leishmania*, a three-fold reduction in flagella velocity and a four-fold reduction in distance dislocated per frame were observed (**Figures 7A, B** and **Supplementary Video 2**). When PF16 was returned to the parasite (complemented cell line), flagella tip movement and velocity were partially restored.

## Discussion

In the Americas, tegumentary leishmaniasis is mainly caused by *Leishmania* species belonging to the *Viannia* subgenus (Grimaldi et al., 1987; Grimaldi et al., 1989). Additionally, two species belonging to this group, *L. braziliensis* and *L. guyanensis*, are potential causative agents of the severe mucocutaneous leishmaniasis (Burza et al., 2018; Silveira, 2019). Major efforts have been applied to unveil the function of more than 8,000 *Leishmania* genes aiming to address the intriguing biology of this parasite (Ivens and Blackwell, 1996). Why different *Leishmania* species cause different clinical outcomes, how does it survive the challenging environmental changes during its life cycle, and other questions remain to be answered. Since 1990, reverse genetics studies using gene disruption through homologous recombination were employed in *Leishmania* spp. to investigate the function of at least 200 genes (Jones et al., 2018). To the best of our knowledge, however, one gene knockout using this approach was obtained in *L. braziliensis* (Atayde et al., 2013), indicating that genome editing in this protozoan remains a challenge. In order to completely replace a gene *via* disruption through homologous recombination, in a diploid organism, at least two rounds of transfection are required using two distinct drug resistance markers, but for multiple copy genes or chromosome number variation, the need for additional transfection rounds makes the process time-consuming and laborious (Bryant et al., 2019). Another limitation of this technique is the requirement of long sequences (>300 nt) to mediate homologous recombination which can be challenging in organisms with important remaining gaps in genome assembly, such as *L. braziliensis* M2903.

CRISPR/Cas9 has facilitated genome editing by allowing complete gene removal in one round of transfection, with no cloning steps. The strategy has already been used to unveil gene function in different *Leishmania* species (Beneke et al., 2017; Martel et al., 2017; Grewal et al., 2019; Shrivastava et al., 2019; Tupperwar et al., 2019; Adaui et al., 2020; Burge and Damianou, 2020; Damasceno and Reis-Cunha, 2020; Damianou et al., 2020; Espada et al., 2021; Turra et al., 2021) with most of the work, so far, been made in the species of the *Leishmania* subgenus and using episomal expression of Cas9. Recently, Adaui et al. (2020) published the first genome editing using CRISPR/Cas9 in *L. braziliensis.* Using LeishGEdit, they were able to knockout the exogenous gene *GFP* and the endogenous genes *HSP100* and *HSP23* in the Peruvian *L. braziliensis* strain PER005, expressing Cas9 and T7 RNA polymerase from an episome.

Herein, we modified the plasmid pTB007 to allow its integration in *L. braziliensis* M2903 β-tubulin locus for the stable expression of Cas9 and T7 RNA polymerase. The pTB007_Viannia cassette was successfully integrated in the β-tubulin locus, and the system has proven to be functional in this cell line. While still untested, it is worth noting that integration of pTB007_Viannia cassette might work in other *Leishmania* spp. belonging to the *Viannia* subgenus due to the high level of identity of the sequences in pTB007_Viannia with at least *L. peruviania*, *L. panamensis*, and *L. guyanensis*.

Consistent with the data shown by Beneke et al. (2017) and Damasceno and Reis-Cunha (2020) in *L. major* stably expressing Cas9 and T7, no significant differences in parasite growth in culture were observed when the Lb C9/T7 cell line was compared with the wild-type strain (Lb WT). Additionally, no differences in metacyclogenesis rates and in infectivity were observed in Lb C9/T7 when compared with Lb WT showing that the stable expression of Cas9 and T7 RNA polymerase does not cause fitness loss in *L. braziliensis*. In this context, it is possible that the differences in growth curve and intracellular survival in *L. braziliensis* expressing Cas9 and T7 RNA polymerase from an episome, reported by Adaui et al. (2020), might be an effect of other factors rather than purely the expression of Cas9. The selection drug on which parasite was kept to avoid plasmid loss and a mild effect of RNAi machinery due to the presence of the episome are possible explanations (Adaui et al., 2020). Another possible reason is variability in expression rates depending on the way the gene was delivered to the parasite for expression (as an episome or as an integrative cassette) and on the genomic region the cassette was integrated. Beneke et al. (2017), for example, reported that while integration in the small subunit ribosomal RNA locus was toxic to the parasite, most likely due to the high rates of transcription of this region, the integration in the β-tubulin locus was well tolerated.

PF16 is a conserved armadillo repeat protein that composes the apparatus of flagella central pair being implicated in maintaining the stability of this structure (Smith and Lefebvre, 1996; Beneke et al., 2017). The *PF16* gene was chosen as a target to test the CRISPR/Cas9 system in Lb C9/T7 since its complete deletion causes a well-characterized and easily detected phenotype of paralysis in *Leishmania* (Beneke et al., 2017). Besides, PF16, which is distributed all over the flagella and flagellar pocket, can be tagged with reporter proteins such as mNG and eYFP (Beneke et al., 2017; Dean et al., 2017; Wang et al., 2020). In the strategy developed by Beneke and Gluenz (2019), two donor cassettes with two different resistance markers are delivered to the cell to mediate DSB repair, resulting in the complete and uniform deletion of CDS without the need for cloning. Herein, using a pair of sgRNAs and only one donor DNA cassette containing a blasticidin resistance gene, complete knockout cell lines were obtained in one round of transfection. In these transfectants, in agreement with the function described in other *Leishmania* species, the removal of *PF16* led to a paralysis phenotype.

Using the Cas9 T7 system, endogenous PF16 was also tagged at the C-terminus with mNG which presented the expected localization, concentrating the mNG signal throughout the entire flagellum and flagellar pocket. This tagged line was purposefully generated using a cassette containing the puromycin resistance marker for further application on in-locus complementation strategy. Using this strategy, we were able to reinsert a tagged copy of PF16 to the original locus from where it was removed in the Lb C9/T7 ΔPF16 cell line. The complementation restored flagellar motility only partially. Divergences between complemented and WT cell line phenotypes are commonly observed. In this case, the addition of a 30-kDa mNG fluorescent protein at the C-terminus might have partially influenced protein function and, thus, the restoration of parasite motility. An impairment in movement was not observed in the PF16 tagged line population, but we cannot guarantee that motility was not affected in this transfectant, since clonal phenotypes were not evaluated. If an impairment in protein function were to be confirmed, an alternative would be to use a smaller tag, such as the myc epitope to overcome this limitation.

This simple strategy for in-locus complementation is an interesting approach, mainly in species with active RNAi machineries that may impair episomal expression (Lye et al., 2010; Adaui et al., 2020). It also avoids possible unforeseeable effects of insertions in other loci under control of stronger promoters, such as the rDNA locus under the control of RNA polymerase I, which may cause overexpression in complemented lines compared with WT.

## Conclusion

Here, we describe for the first time the integration of a Cas9 and T7 RNA polymerase expression cassette in the *L. braziliensis* M2903 β-tubulin locus, allowing the stable and constitutive expression of this machinery without the need for continuous drug selection for plasmid maintenance. Importantly, this was achieved without fitness loss as measured by growth rates, metacyclogenesis, and infectivity in this novel Lb C9/T7 cell line. In addition, this strain is compatible with gene editing utilizing primers directly generated by LeishGEdit. Furthermore, this work describes an in-locus complementation strategy, which is simple and can circumvent the traditional episomal complementation strategy, which in *L. braziliensis* has the potential to be extremely challenging due to the active RNAi machinery in this species. The system presented herein is a straightforward, time-saving strategy that can be used to unveil the function of coding and non-coding elements of *L. braziliensis* genome.

## Data Availability Statement

The original contributions presented in the study are included in the article/**Supplementary Material**. Further inquiries can be directed to the corresponding author.

## Author Contributions

This study was conceptualized by CE, SU, AA-W, and EG and conducted under the supervision and guidance of EG, AC, and SU in their laboratories. CE, JQ, and AA-W performed the experiments. CE, AA-W, JQ, and MC performed the data analysis. TB developed the original plasmids and the editing system together with EG. LL idealized the in-locus complementation strategy. JQ, AA-W, MC, TB, and LL gave valuable assistance during the experiment. CE, JJ, MC, and SU wrote the manuscript. All authors contributed to the article and approved the submitted version.

## Funding

This work was supported by Fundação de Amparo à Pesquisa do Estado de São Paulo (FAPESP 2016/23405-4, 2018/25299-2, 2020/00087-2, 2018/14398-0, 2020/00088-9, 2016/00969-0, 2015/09080-2). SU is the recipient of a senior researcher scholarship from the Brazilian National Council for Scientific and Technological Development (CNPq 306971/2018-6). AC also acknowledges Coordenação de Aperfeiçoamento de Pessoal de Nível Superior – Brasil (CAPES) – Finance Code 001 and CNPq (305775/2013-8). AA-W acknowledges Fundação para a Ciência e a Tecnologia for funds to GHTM (UID/04413/2020). TB was supported by the Medical Research Council (15/16_MSD_836338), and EG is a Royal Society University Research Fellow and supported through the WCIP core Wellcome Centre Award No. 104111/Z/14/Z.

## Conflict of Interest

The authors declare that the research was conducted in the absence of any commercial or financial relationships that could be construed as a potential conflict of interest.

## Publisher’s Note

All claims expressed in this article are solely those of the authors and do not necessarily represent those of their affiliated organizations, or those of the publisher, the editors and the reviewers. Any product that may be evaluated in this article, or claim that may be made by its manufacturer, is not guaranteed or endorsed by the publisher.

## Supplementary Material

Supplementary Table 1 List of the primers used in this study.

Supplementary Video 1 Evaluation of parasite motility in parental Lb C9/T7 (C1), knockout Lb C9/T7 ΔPF16 (C2) and complemented Lb C9/T7 PF16::mNG (C3) lines. Parasites were seeded in microscopy slides and parasite movement was qualitatively observed. Scale bar 5 µm.

Supplementary Video 2 Trajectory of flagella tip in parental Lb C9/T7 (C1), knockout Lb C9/T7 ΔPF16 (C2) and complemented Lb C9/T7 PF16::mNG (C3) lines. Parasites were seeded in microscopy slides and left to decant for at least 20 minutes before acquisition to minimize the effect of fluid dislocation. Videos of living cells were acquired at 20fps and used to determine velocity and dislocation rates of flagella tip upon tracking using FIJI (35). Scale bar 10 µm.
